# Engineering Plasma–Liquid Microdischarge Systems for Direct N_2_‑to-NH_3_ Conversion at Ambient Conditions

**DOI:** 10.1021/acssuschemeng.5c13108

**Published:** 2026-03-14

**Authors:** Marco Francesco Torre, Lavanya Veerapuram, Francesco Tavella, Chiara Genovese, Siglinda Perathoner, Federica Torrigino, Pierdomenico Biasi, Gabriele Centi, Claudio Ampelli

**Affiliations:** † Department of Chemical, Biological, Pharmaceutical and Environmental Sciences (ChiBioFarAm), 18980 University of Messina, ERIC aisbl and CASPE/INSTM, Viale Ferdinando Stagno d’Alcontres 31, 98166 Messina, Italy; ‡ Basic Research Department, 726912 Casale SA, Via G. Pocobelli 6, 6900 Lugano, Switzerland

**Keywords:** sustainable ammonia synthesis, plasma−liquid interaction, nitrogen fixation, microdischarge, nonthermal plasma

## Abstract

Ammonia (NH_3_) can be synthesized directly from N_2_ and H_2_O using plasma *micro*-discharges formed at the water–electrode interface, offering a promising alternative to both conventional electrocatalysis and nonthermal plasma processes. However, discharge performance and stability are strongly affected by device engineering. This study reports the development and engineering of a hybrid electrochemical device that integrates a *micro*-plasma cathode for sustainable NH_3_ production under ambient temperature and pressure. Solvated electrons generated through plasma–liquid interactions, particularly within interfacial aerosol microdroplets, act as highly reducing species, eliminating the need for catalysts or external chemical reagents. The effects of the plasma–liquid gap, gas feed flow rate, discharge current, and cathode inner diameter on NH_3_ yield are systematically investigated. Optimizing these factors enables Faradaic efficiency exceeding 70% and significantly enhances the instantaneous N_2_-to-NH_3_ yield, outperforming previously reported plasma–liquid systems. These findings highlight the importance of system engineering optimization for advancing sustainable plasma-assisted nitrogen fixation and for progressing toward industrial scale-up.

## Graphical abstract

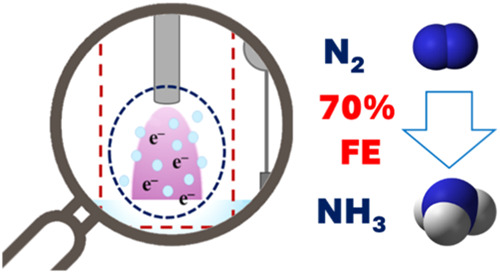

## 1 Introduction

Ammonia (NH_3_) is the leading volume chemical (approximately 150 million metric tons in 2024), serving as the basis for fertilizer production (around 70%), while the remainder is used for various industrial applications, such as plastics, explosives, and synthetic fibers.^1,2^ Increasing interest is being shown in its use as an energy or H_2_ carrier, which is forecast to result in rapid demand growth in the future.^3^ However, this application requires a change in the modalities of production, from the current mega-scale plants, based nearly exclusively on the Haber-Bosch (H–B) process, to new, distributed (smaller-scale) plants that use renewable energy for the crucial step of H_2_ production (mainly by electrolysis), i.e., green NH_3_ production.^2^

Global NH_3_ production accounts for ∼2% (8.6 EJ) of total final energy consumption, with around 40% of this energy input associated with the use of raw materials (primarily fossil fuels, mainly methane) as hydrogen sources.^4^ The current trends in decarbonisation and electrification of chemical production prompt the NH_3_ industry to seek alternative solutions to the H–B process.^5,6^ The current focus is on H_2_ production via water electrolysis, followed by a thermocatalytic step such as the H–B process, even if milder pressures are required. This route is highly energy-intensive, and the coupling between the electro- and thermo-catalytic steps is not optimal.^7^ For distributed production, there is growing interest in alternative technologies, based on renewable energy, that enable the direct synthesis of NH_3_ from N_2_, rather than a two-step process via molecular H_2_, which also introduces several thermodynamic limitations.^8,9^

Two main directions are currently under investigation: (i) the direct electrocatalytic reduction of N_2_ to NH_3_ (NRR)^8^ and (ii) the use of nonthermal plasma (NTP) with or without the presence of a catalyst.^6^ Despite considerable research interest, the performance of these methods remains insufficient for practical application.^10–13^ There is thus growing interest in exploring alternative strategies, including combining these approaches to exploit potential synergies.

Among these directions, plasma–liquid (P–L) systems have shown promise in recent years for green N_2_ fixation using water (H_2_O) as the hydrogen source.^14–16^ Researchers have used various configuration designs, including the “plasma generated over liquid” setup, which involves generating plasma above the liquid surface. In this configuration, the plasma not only provides activated reactive species (vibrationally and electronically excited species, electrons, ions, etc.) but also acts as an essential part of the electrical circuit.^17–19^ The *micro*-discharge between the plasma jet electrode and the water (acting as electrolyte and in contact with the counter-electrode; see later) generates a current that closes the electrochemical circuit. Other systems are “*in*-liquid plasma”, where plasma is generated directly within the liquid by ionizing a gas. These systems are often integrated into hybrid devices, where the plasma generates nitrogen oxides (NO_*x*_), which are then electrocatalytically reduced to NH_3_.^20,21^ Other systems, called “remote plasma”, use plasma jets to deliver activated species into the bulk liquid. In contrast to systems where plasma is generated in contact with water, the plasma generation, in this case, is independent of the primary electrical circuit.^22–24^

Hawtof et al.^17^ employed a hybrid plasma DC electrolysis system. They demonstrated that it can be used as a tool to study NH_3_ formation without a catalytic material, achieving high Faradaic efficiencies (up to 100%) and an integrated productivity of 0.44 mg h^–1^ at ambient temperature and pressure, utilizing N_2_ and H_2_O. Ramoy et al.^18^ used a system to synthesize NH_3_ without catalysts. A stable N_2_ plasma was generated inside bubbles in water (*in*-liquid plasma) even when the water surface itself acted as the cathode for the DC discharge. A maximum NH_3_ productivity of about 1.06 mg h^–1^ was indicated. Although these studies have demonstrated the feasibility of the approach, an engineering analysis of the system optimization, as a prodrome to scale-up and industrialization, is not available, nor are the performance metrics sufficiently linked to mechanistic aspects, particularly regarding the reaction location. While previous authors have indicated that the reaction occurs at the discharge interface with water, Pattyn et al.,^19^ using a DC-powered N_2_ plasma-electrolysis system, suggested that NH_3_ is primarily formed in the gas phase and with greater selectivity at low currents (<5 mA). Different approaches in combining NTP and electrochemistry have thus been investigated. Still, the key factors controlling performance and their links to the mechanistic aspects of NH_3_ formation remain poorly understood.

We present a systematic engineering study of a hybrid device incorporating a *micro*-plasma cathode that discharges into an aqueous electrolyte for the sustainable direct synthesis of NH_3_ at ambient conditions, utilizing N_2_ and H_2_O directly. The N_2_ molecules are activated (vibrationally and electronically) in the NTP. Simultaneously, the energetic (hot) electrons generated in the plasma *micro*-discharge interact with H_2_O, creating hydrogen species that react with the activated N_2_ molecules to form NH_3_ or ammonium ions (NH_4_^+^).

The process is highly dependent on operational parameters/aspects, making it essential to analyze their impact on performance while also providing indications of mechanistic features. These aspects were investigated by optical emission spectroscopy (OES), which provides indications on the types of species formed in the *micro*-discharge. In this hybrid plasma-electrolytic device, we have systematically analyzed the influence of various key operational parameters: (i) the distance between the capillary electrode tip, generating the plasma microjet, and the liquid electrolyte surface (gap distance), (ii) the gas flow rate, and (iii) the plasma electrode inner diameter (ID). The study also includes various additional aspects regarding system engineering and optimization, suggesting directions for advancing sustainable plasma-assisted nitrogen fixation technologies and for moving toward scale-up for industrialization.

## 2 Methods

The hybrid device used in the tests, incorporating a plasma *micro*-discharge cathode, is schematically illustrated in Figures 1 and S1. This system utilizes a *micro*-plasma generated in the gas-phase volume gap between a stainless-steel capillary tube and the electrolyte solution surface. The plasma works as the cathode, while a platinum plate electrode immersed in the solution serves as the anode for the oxygen evolution reaction (OER):

1 $$2H_{2}O\to 4e^{-}+4H^{+}+O_{2}↑$$

**Figure 1:**
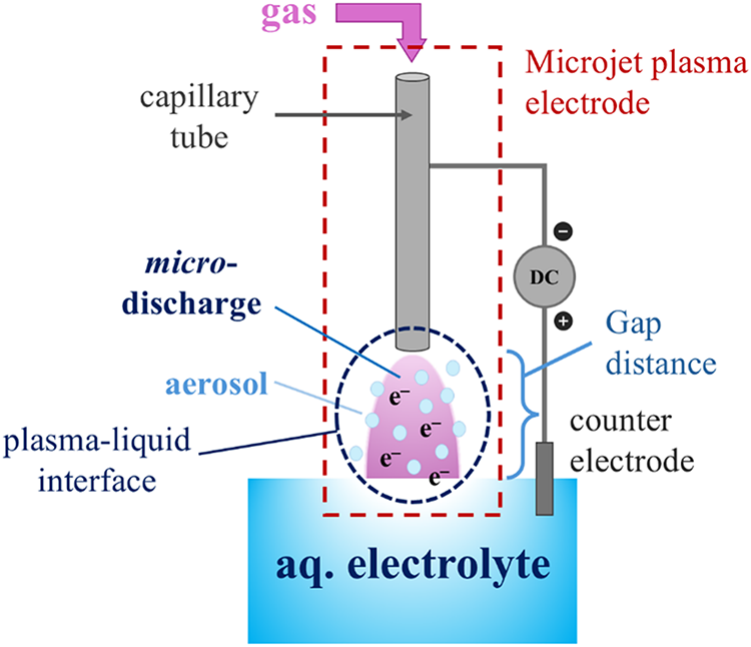
General schematic representation of the hybrid plasma-electrolytic system.

The electrolyte solution was 0.25 mM sulfuric acid (H_2_SO_4_) in Milli-Q water; this acidic solution supplies protons (H^+^) for N_2_ reduction, captures the synthesized NH_3_ as NH_4_^+^, and maintains sufficient electrical conductivity due to the presence of H^+^ ions.

The system operates from a DC power supply and employs galvanostatic control via a series ballast resistor (*R*_b_). A second resistor (*R*_i_) is used to determine the current by monitoring the voltage drop across it. The voltage between the two electrodes is measured using a high-voltage probe. Materials and further setup details are provided in the Supporting Information (SI).

To ensure precise analysis of the produced NH_3_, a blank test (BT) was conducted before each experiment to detect any pre-existing NH_3_ in the experimental atmosphere. Each BT consisted of purging the electrolyte solution with N_2_ at a flow rate of 50 mL min^–1^ for 15 min, simulating the degassing (saturation) procedure used in the main tests. Additionally, to detect any NH_3_ formed in the gas phase, an acid trap containing the same solution used as the electrolyte was placed at the cell’s gas outlet. Analysis of the trap solution at the end of each test never detected NH_4_^+^. This confirms that, for such P–L systems employing an acidic solution (pH < 5), all synthesized NH_3_ is directly trapped in the bulk acidic electrolyte solution as NH_4_^+^.

For additional security against external contamination, all experiments and analyses were conducted in an isolated box with a purified, controlled atmosphere that prevents NH_3_ contamination from external sources. This experimental protocol ensures that no accidental external contamination by NH_3_ occurs during testing. Additionally, we continually monitored the absence of N-containing species in the electrolyte using ion chromatography and UV–vis analysis. These analyses confirmed that none of the materials involved in the hybrid system (e.g., electrodes, cell components, vessel) introduces nitrogen-based contaminants. This was further verified by tests using Ar instead of N_2_ as the gas for generating the *micro*-discharge in a N_2_-free atmosphere. The absence of any detectable NH_3_ species in these control BTs clearly proves that NH_3_ formation is not associated with N-contaminants and their transformation induced by NTP, including potential contaminants in the gas feed to the microjet plasma electrode.

Furthermore, switching the gas flow from N_2_ to Ar (and back) confirmed that NH_3_ forms only when a N_2_ flow is present. We consider this protocol, together with the other BTs indicated above, to be more robust and preferable to using isotopically labeled N_2_. The latter, due to costs, cannot conduct continuous extended tests and, additionally, does not allow analysis of the absence of labeled NO_*x*_. We performed occasional tests with labeled N_2_ to confirm the validity of our experimental protocol. However, we adhere to the rigorous protocol outlined above, which we consider a preferable standard quality check.

A high-purity N_2_ cylinder was used in all tests. The potential presence of NH_3_ or NO_*x*_ contaminants in the N_2_ flow was checked in the preliminary tests by constantly monitoring the absence of the corresponding species in the electrolyte, without applying the discharge voltage. In addition, OES measurements (Figures 4 and S5) excluded the in situ formation of NO_*x*_ species in the *micro*-discharge gas phase. The experimental protocol thus ensures that the detected NH_3_ is formed directly from N_2_, rather than from accidental contaminants or the reduction of NO_*x*_ species present in the feed gas.

For convenience, we refer to the product as “NH_3_“. However, all calculations and analyses will consider the combined production of NH_3_ in the gas phase and NH_4_^+^, in the liquid phase as the relevant species. Special analytical attention was given to the analysis of other possible generated species, such as hydrogen peroxide (H_2_O_2_), hydrazine (N_2_H_4_), and hydroxylamine (NH_2_OH). We have no evidence for their formation under our conditions, confirmed through multiple analytical techniques, including ^1^H NMR. We also tested NH_3_ formation using different analytical techniques^16^ to verify the correctness of the analytical procedure, with details reported in the SI. The only other product detected, H_2_, formed during the tests, was measured using gas chromatography, as described in the SI.

The SI also provides details on data acquisition and processing, including the methods used to determine the Faradaic Efficiency (FE), the integrated NH_3_ overall productivity in 1 h (PR), and the instantaneous N_2_-to-NH_3_ (NH_4_^+^) yield (YI). The protocol used to calculate energy consumption is also described. Reproducibility of the results was determined by performing multiple tests, with a typical variation of ± 5%. Once the *micro*-discharge was stabilized, consistent performance was maintained over an 8-h test period. Furthermore, the system demonstrated high reliability over several hours of cumulative operation using the same stainless-steel capillary cathode, with no observable degradation in discharge behavior and no need for surface conditioning or polishing. No change in electrolyte pH was observed over the investigated time scale (within ± 0.1%).

## 3 Results

Complex physical, chemical, and electrochemical processes characterize the plasma-liquid interface.^25,26^ In the interfacial region, there is a dynamical coupling between the gaseous plasma and liquid phase through gas-phase ionization and excitation, liquid evaporation, diffusion of species across the interface, electric-field dissipation and charge transfer, and reactive chemistry within each phase. Highly reactive solvated electrons generated inside the NTP are injected into the bulk water (electrolyte) by the plasma.^14,27,28^ Due to the short lifetimes of these solvated electrons, they have a short diffusion path in the bulk liquid, of the order of a few nm.^29,30^ For this reason, the generation of an aerosol at the interface, as outlined in Figure 1, resulting from the *micro*-discharge’s impact on the aqueous electrolyte, plays a crucial role in increasing the interfacial area between the gas plasma and the liquid phase. This phenomenon, often referred to as nanoscale plasma-activated aerosol (PAA),^31,32^ can significantly enhance mass and energy transfer, although it has not been explicitly investigated for N_2_-to-NH_3_ conversion.

To the best of our knowledge, the operative parameters influencing PAA generation and their impact on the rate of NH_3_ synthesis from N_2_ and H_2_O in a hybrid plasma-electrolytic system have not been investigated. We thus report here the role of key engineering parameters in NH_3_ production, focusing on optimizing the Faradaic efficiency (FE), the N_2_-to-NH_3_ (NH_4_^+^) instantaneous yield (YI), and the overall productivity (PR). We identified a series of key parameters that significantly impact the properties and behavior of this interfacial region:

1. Gap distance: the distance between the capillary tube and the surface of the electrolyte solution.
2. N_2_ flow rate: the rate of nitrogen gas supplied to sustain the plasma; it influences both plasma stability and mass transfer.
3. Capillary tube inner diameter (ID) determines the velocity and confinement of the gas jet, and thus the characteristics of the *micro*-discharge.
4. Electrolyte solution saturation time: the pretreatment duration during which N_2_ is bubbled into the solution before plasma activation.
5. Applied current: a key parameter controlling the intensity and related to the energy input of the *micro*-discharge.
6. Stirring of the electrolyte solution: it can modulate mass transport within the liquid phase and affect aerosol dispersion.

### 3.1 Effect of Gap Distance

The gap distance between the end of the microjet plasma electrode and the aqueous electrolyte surface is a critical parameter for plasma ignition in hybrid plasma-liquid systems,^33^ as it influences the *breakdown voltage* and facilitates the efficient injection of activated species into the bulk liquid. While the gas flow from the capillary tube perturbs the surface of the aqueous electrolyte, only a relatively small concave zone is created in our experimental conditions. In contrast, when a stable *micro*-discharge is created, a distinct aerosol of nanodroplets forms due to the combined effects of local heating (although the overall temperature of the aqueous electrolyte does not change significantly, remaining around 35 °C) and, especially, plasma-induced aerosol generation.^34^

We initially performed experiments by varying the gap distance between the capillary tube and the electrolyte solution (Figure 2a). The gap was varied from 1 to 3 mm, while other parameters were held constant. All the set parameters are reported in Figure 2a.

**Figure 2:**
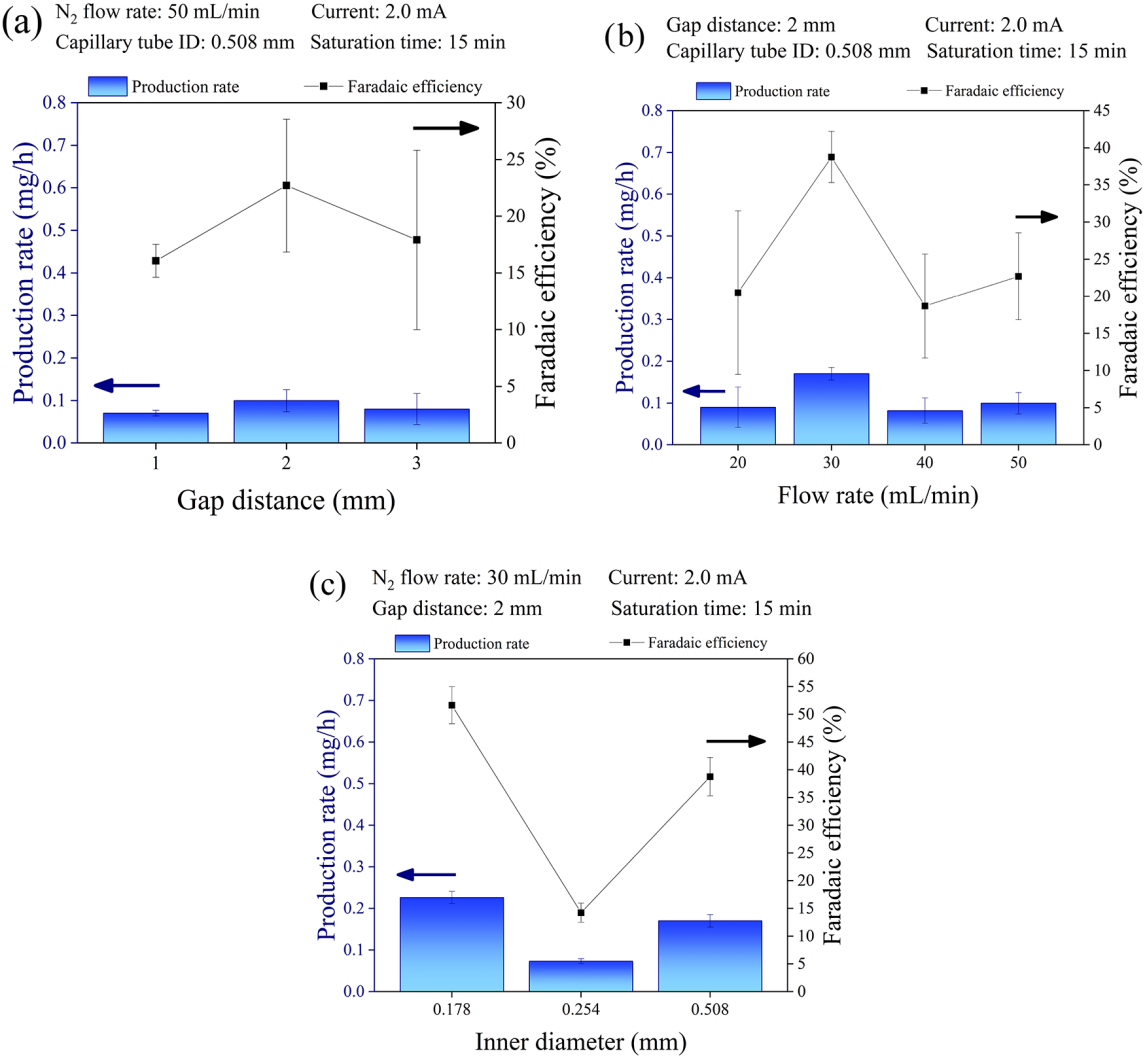
NH_3_ Faradaic efficiency and production rate in the plasma-electrolytic system under various operational conditions. The effects of (a) the gap distance between the microjet plasma and the electrolyte solution surface, (b) the N_2_ flow rate through the capillary of the microjet plasma electrode, and (c) the capillary tube inner diameter (ID) of the microjet plasma electrode are shown. Complete data sets are provided in the SI (Tables S1–S3).

Figure 2a illustrates the effect of varying the gap distance between the capillary tube and the electrolyte surface on the FE and PR of NH_3_ synthesis. While a shorter distance (1 mm) might appear optimal for reducing plasma *breakdown voltage* and energy consumption (discussed later) and minimizing the travel distance of activated species injected into solution, the results demonstrate that the optimal distance for our system to enhance both FE and PR was 2 mm. This is consistent with the mechanisms of aerosol generation and its critical role in determining productivity. When the gap distance is too short, the volume of the interface aerosol is low. In addition, the electrode may come into contact with the aqueous solution due to water droplets spreading from the bulk liquid by both the *micro*-plasma and the gas flow, ultimately causing plasma failure, as already observed by Luo et al.^20^ for a similar plasma-electrolytic system. Conversely, increasing the gap to 3 mm resulted in plasma ignition at higher voltages, as expected under *Paschen’s law*.^33^ This larger gap also increased instability during operation, resulting in lower FE and PR, and reduced reproducibility. Based on these observations, we selected a gap distance of 2 mm for all subsequent experiments.

### 3.2 Effect of N_2_ Flow Rate

Given that nitrogen species involved in NH_3_ synthesis exclusively originate from plasma-activated N_2_, controlling the flow rate significantly influences NH_3_ production. Thus, the effect of the N_2_ flow rate on FE and PR was investigated by varying it from 20 to 50 mL min^–1^, as shown in Figure 2b. All other operational parameters were maintained at the same values as the previous set of experiments. All the set parameters are reported in Figure 2b.

As shown in Figure 2b, the NH_3_ PR and FE do not exhibit a linear correlation with the increasing gas flow rate. Initially, the NH_3_ PR increases with the N_2_ flow rate, reaching a peak at 30 mL min^–1^. Interestingly, increasing the flow rate beyond 50 mL min^–1^ did not result in a further increase in FE and PR. This observation suggests that, for these specific operating conditions (gap distance, flow rate, and capillary tube ID), a flow rate of 30 mL min^–1^ is optimal. This behavior, with a maximum at an intermediate flow rate, is consistent with the aerosol generation mechanism,^26^ particularly the near-surface gas dynamics, i.e., the sticking coefficient of gas-phase electrons with nanodroplets. The observed trend of maximum NH_3_ concentration at intermediate gas flow rates is also consistent with the results of Wang et al.^35^ Furthermore, this behavior is not unique to NH_3_ synthesis, as similar trends have been reported by Zhang et al.^36^ for plasma-assisted CO_2_-to-C_2_O_4_^2–^ (oxalate) and H_2_O-to-H_2_O_2_ (hydrogen peroxide) conversion in pulsed-discharge P–L systems. At this point, the system appears to achieve a balance between maximizing the activation of reactive nitrogen species and efficiently injecting electrons into the electrolyte solution.

### 3.3 Effect of Capillary Tube Inner Diameter (ID)

The effect of the capillary tube inner diameter (ID) on NH_3_ PR and FE was evaluated using three different nozzle sizes: 0.178, 0.254, and 0.508 mm, as shown in Figure 2c. All other operational parameters were maintained at the same values as the previous set of experiments. All the set parameters are reported in Figure 2c.

As shown in Figure 2c, no linear correlation was observed in PR and FE when decreasing ID. The smallest ID (0.178 mm) consistently yielded the highest PR and FE. Notably, the 0.178 mm capillary tube significantly improved *micro*-plasma stability compared to the previously used 0.508 mm capillary tube. This behavior can be attributed to the changing flow velocity (*Q* = *A* × *v*) of N_2_ (including all activated species and electrons generated by the plasma) at the capillary tube exit. Maintaining a constant volumetric flow (*Q*), reducing the cross-sectional area (*A*) resulted in a higher flow velocity (*v*). This increased velocity significantly improved the PR and FE for NH_3_ synthesis. This is consistent with the interaction of gas-phase electrons with nanodroplets, as discussed above, indicating that electron density and energy vary with interface characteristics and microfluid dynamics, i.e., the manner in which reactive species impinge on the nanodroplet surface. On the other hand, it is challenging to explain the experimental observations through different mechanisms.

It may be noted that the presence of the discharge and a short gap (around 2 mm) prevents characterization of the aerosol using methods such as microscopy, photoacoustic spectroscopy, dynamic light scattering, and others.^37^ On the other hand, we made several attempts to increase the concentration of nanodroplets by introducing an external flow of fine water droplets generated by a nebulizer. However, this additional flow destabilized the discharge (Figure S4), preventing the acquisition of reliable data and rendering the parameter optimization performed so far ineffective. Therefore, the generation and role of aerosols can only be inferred from indirect evidence, although these inferences are supported by results reported by other authors using different reactor configurations.^31,32^

To verify the enhancement effect of microdroplets on the NH_3_ PR, we configured a dielectric barrier discharge (DBD) reactor (Figure S7) operating with a continuous aerosol flux (see Section 3.9 for details).

### 3.4 Effect of Electrolyte Saturation Time

The effect of electrolyte saturation time on NH_3_ PR and FE was evaluated by varying the N_2_ purging time of the acidic electrolyte solution from 15 to 60 min, as illustrated in Figure 3a. All other operational parameters were maintained at the same values as the previous set of experiments. All the set parameters are reported in Figure 3a.

**Figure 3:**
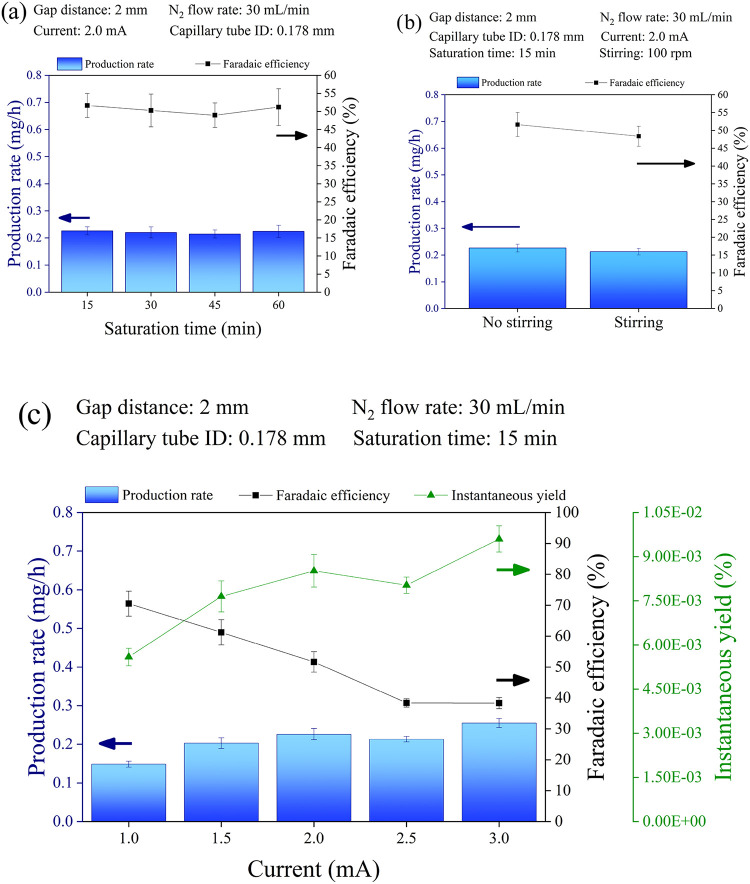
NH_3_ Faradaic efficiency, production rate and instantaneous yield in the plasma-electrolytic system under various operational conditions. The effects of (a) the electrolyte saturation time, (b) the stirring of the electrolyte solution, and (c) the plasma discharge current are shown. Complete data sets are provided in the SI (Tables S4, S5a–b, S6).

To investigate the effect of various electrolyte saturation times on NH_3_ PR and FE, we first determined the minimum time required to thoroughly saturate the volume of electrolyte solution used in each test run. The latter was fixed to 20 mL (see *Section 1.3* in the SI for more details).

To establish a baseline, we first determined the minimum time required to achieve complete saturation of the 20 mL electrolyte solution used in each experiment (SI *Section 1.3*). Applying *Henry’s Law* (eq 2) and utilizing the *Henry’s Law constant* (*H*_*s*_^*cp*^) for N_2_ in H_2_O at 298.15 K (6.5 × 10^–4^ mol L^–1^ atm^–1^),^38^ we calculated the equilibrium N_2_ concentration (solubility) at the onset of N_2_ purging (*t* = 0). Given the initial N_2_ partial pressure (*p*) of approximately 0.78 atm (corresponding to the atmospheric N_2_ concentration), the calculated N_2_ solubility is 5.1 × 10^–4^ mol L^–1^. Our calculations (SI *Section 3*) demonstrated that, with an N_2_ flow rate of 10 mL min^–1^, complete electrolyte saturation can be achieved in under 1 min. Consequently, a standardized saturation time of 15 min at 50 mL min^–1^ was confirmed to ensure full saturation of the 20 mL electrolyte solution and the 30 mL headspace of the electrolytic cell. This extended saturation period also allowed for the gradual displacement of air within the headspace, ultimately resulting in a nearly 100% N_2_ atmosphere.

2 $$c=H_{s}^{\mathrm{cp}}\times p$$

The results presented in Figure 3a reveal that increasing the electrolyte solution saturation time beyond the standardized 15 to 60 min yielded no substantial change in NH_3_ PR or FE. This indication is in agreement with the results of Hawtof et al.,^17^ which indicated that N_2_ availability in the electrolyte solution is not the primary limitation for NH_3_ production. This result is also consistent with a reaction mechanism confined to the gas-phase zone, not influenced by the N_2_ concentration in the bulk of the electrolyte, but limited by the N_2_ concentration within the aqueous droplets. The dominance of the hydrogen evolution reaction (HER), discussed in detail later, can explain this observation. Therefore, exploring alternative solvent systems, such as organic solvents known to exhibit higher N_2_ solubility,^38,39^ may offer a pathway to improved NH_3_ PR and FE. Nevertheless, it is crucial to acknowledge and address potential side reactions associated with the synthesis or degradation of these organic solvents.^40^

### 3.5 Effect of Electrolyte Solution Stirring

If the locus of NH_3_ synthesis is the aqueous region at the *micro*-discharge,^41^ a significant effect of stirring the electrolyte solution is expected. The effect of the electrolyte solution stirring on NH_3_ PR and FE was evaluated by stirring the solution during plasma operation at 100 rpm. All other operational parameters were maintained at the same values as the previous set of experiments. All the set parameters are reported in the inset of Figure 3b.

Note that the solution’s stirring speed is critical for maintaining plasma stability during both initiation and operation. To ensure stable plasma while assessing the impact of stirring on PR and FE in NH_3_ synthesis, a stirring speed of 100 rpm was selected, as it was the highest speed that provided comparable (with previous tests of this work) plasma stability throughout the test period. In fact, increasing the stirring speed beyond 100 rpm made it impossible to maintain acceptable stability of the *micro*-discharge during both ignition and testing.

While the previously investigated operational parameters (gap distance, N_2_ flow rate, and capillary tube ID) were found to have a stronger influence on plasma-generated species (e.g., activated nitrogen species and solvated electrons), we hypothesized that stirring the electrolyte solution during plasma operation could enhance the mass transport of H^+^ from the bulk solution to the area beneath the plasma *micro*-discharge. This includes both the initial H^+^ concentration provided by H_2_SO_4_ and the additional H^+^ generated by the OER (eq 1) at the anode. Improved H^+^ transport by stirring the electrolyte solution is expected to increase the availability of H^+^ at the P–L interface, potentially leading to higher NH_3_ FE and PR.

Before plasma ignition, the H^+^ concentration at the P–L interface is equal to that in the bulk solution. Upon plasma ignition, the concentration of solvated electrons rapidly increases due to the injection of plasma-generated electrons into the liquid phase. These solvated electrons initially reduce H^+^ to hydrogen radicals, as discussed in detail later, thereby depleting H^+^ at the P–L interface. Stirring the electrolyte solution is therefore expected to improve the mass transport of H^+^ from the bulk solution to the P–L interface, mitigating local H^+^ depletion.

The results reported in Figure 3b indicate that stirring the aqueous electrolyte solution has a slightly adverse effect on both PR and FE, contrary to the initial assumption. This observation is consistent with previous reports.^42^ Delgado et al. reported that minimum fluid-dynamic conditions, i.e., a liquid flow velocity of 10^2^–10^4^ m s^–1^, are required to replenish the interface of a P–L system.^42^ Anyway, the stirring would increase the P–L interface and thus a positive, rather than negative, effect could be expected. On the other hand, stirring would reduce aerosol generation by minimizing the electrospray effect, i.e., the formation of a Taylor cone and subsequent breakup into charged droplets when a high voltage is applied to a liquid.^25^ The results in Figure 3b thus further confirm the key role of aerosol in N_2_-to-NH_3_ synthesis.

### 3.6 Effect of Discharge Current

The effect of the discharge current on NH_3_ PR and FE was evaluated by varying the galvanostatically controlled operational current from 1.0 to 3.0 mA (Figure 3c). All other operational parameters were maintained at the same values as the previous set of experiments. All the set parameters are reported in the inset of Figure 3c.

The high-voltage generator controlled the plasma discharge current galvanostatically. The current value was set before plasma ignition and remained constant throughout plasma operation. We emphasize that the plasma-generated current was the sole electrical power supplied to the system, and no external electrical bias was applied.

Increasing the plasma discharge current increases the density of plasma-generated electrons, which can excite N_2_ or inject electrons into the electrolyte solution. According to *Faraday’s first law*, an increase in NH_3_ productivity is expected. However, experimental data (Figure 3c) show that the PR rises from 1.0 to 2.0 mA discharge current, then increases slowly to 3.0 mA. Parallel to this, the FE, which is 70.5% at the minimum discharge current, rapidly drops to approximately 40% at higher discharge current values. Note that the N_2_-to-NH_3_ (NH_4_^+^) instantaneous yield (YI) instead increases with the discharge current.

The PR specifies the device’s average production rate. At the same time, the YI is calculated as the ratio of NH_4_^+^ (NH_3_) produced to the amount of injected N_2_ at the selected flow rate (see SI for definition). Thus, PR provides a global indication of the amount of NH_3_ produced under specific conditions in the device. At the same time, YI is proportional to the ratio between formed NH_4_^+^ and N_2_ feed to the device. When the amount of the latter is fixed, as in the tests shown in Figure 3c, YI is indicative of the instantaneous reaction rate. FE is instead indicative of the selectivity for producing NH_3_ over H_2_, the only other product observed in these tests. As the discharge current increases, the number of electrons generated in the plasma also rises.

The reaction pathway competing with NH_3_ synthesis is the hydrogen evolution reaction (HER), which occurs either through the recombination of solvated electrons (e_(aq)_^–^):

3 $$2e_{\left(\mathrm{aq}\right)}^{-}+2H_{2}O_{\left(l\right)}\to H_{2}↑+\;2{\mathrm{OH}}_{\left(\mathrm{aq}\right)}^{-}$$

4 $$2e_{\left(\mathrm{aq}\right)}^{-}+2H_{3}O_{\left(\mathrm{aq}\right)}^{+}\to H_{2}↑+\;2H_{2}O_{\left(l\right)}$$

or through the recombination of hydrogen radicals (H^•^):

5 $$2e_{\left(\mathrm{aq}\right)}^{-}+2H_{\left(\mathrm{aq}\right)}^{+}\to {2H}_{\left(\mathrm{aq}\right)}^{•}$$

6 $$2H_{\left(\mathrm{aq}\right)}^{•}\to H_{2}↑$$

with the reactions depicted in eqs 4 and 5 being dominant under our acidic conditions (0.25 mM H_2_SO_4_). However, the H-type species are also involved in the mechanism of NH_3_ synthesis, as commented later. Thus, it may not be expected that there is a substantial decrease in FE (which is 70.5% at a discharge current of 1.0 mA) with a parallel increase in YI on increasing discharge current, and the flux of plasma-generated electrons. On one side, the increase in discharge current also increases the flux of activated N_2_ molecules (see later), consistent with the observed increase in YI. However, this does not explain the decrease in FE.

Another possible mechanism of H_2_ production is via electron-impact dissociation of H_2_O in the nanodroplets of the plasma-induced aerosol. Toth et al.^43^ reported an electron-impact dissociation of H_2_O vapor in a DC P–L system following eq 7, with the generated H^•^ (and hydroxyl radical (OH^•^)) producing then H_2_ following eq 6:

7 $$e^{-}+H_{2}O\to e^{-}+H^{•}+{\mathrm{OH}}^{•}$$

However, the mechanism is more effective with water nanodroplets in the plasma-induced aerosol than with H_2_O vapor molecules, and the increased aerosol generation with increasing discharge current. Thus, on one side, the aerosol promotes the interfacial area, the rate between activated N_2_ molecules and the H^•^ produced by reaction of e_(aq)_^–^ with H_2_O, but at the same time also the side reaction of H_2_ production. This explains the increased YI, while a decrease in FE occurs with increasing the discharge current. There are thus two main mechanisms to form the hydrogen radicals, which play an essential role in directly reducing N_2_ to NH_*x*_ species: the first is by reaction of electrons (generated by NTP) with protons at the discharge-nanodroplets interface, and the second by water homolysis, which simultaneously generates hydroxyl radicals. The latter are very reactive and generate NO_*x*_ species, but mainly in the bulk electrolyte, as commented later. The first mechanism for hydrogen radical generation is instead the dominant one for producing ammonia.

There are thus complex chemical phenomena at the interfacial P–L interface, and from here, a complex dependence on device configuration and operational parameters.

### 3.7 Optical Emission Spectroscopy Results

To gain deeper insight into the intricate chemistry at the P–L interface in our system, experiments were performed during plasma operation, monitoring species formed by optical emission spectroscopy (OES) under different test conditions. Figures 4 and S6 show the OES spectra obtained. Note that no peaks are observed in the 200–280 nm region, where NO_*x*_ species, if present, are expected to be visible.^19,35^ In particular, the NO γ-system (A^2^∑^+^ → X^2^∏) and β-system (B^2^∏ → X^2^∏) have prominent emission bands in this range. The absence of bands in the 200–280 nm region thus clearly indicates that, under our experimental conditions, neither NO_*x*_ species is present as a contaminant nor is generated in the gas phase.

**Figure 4:**
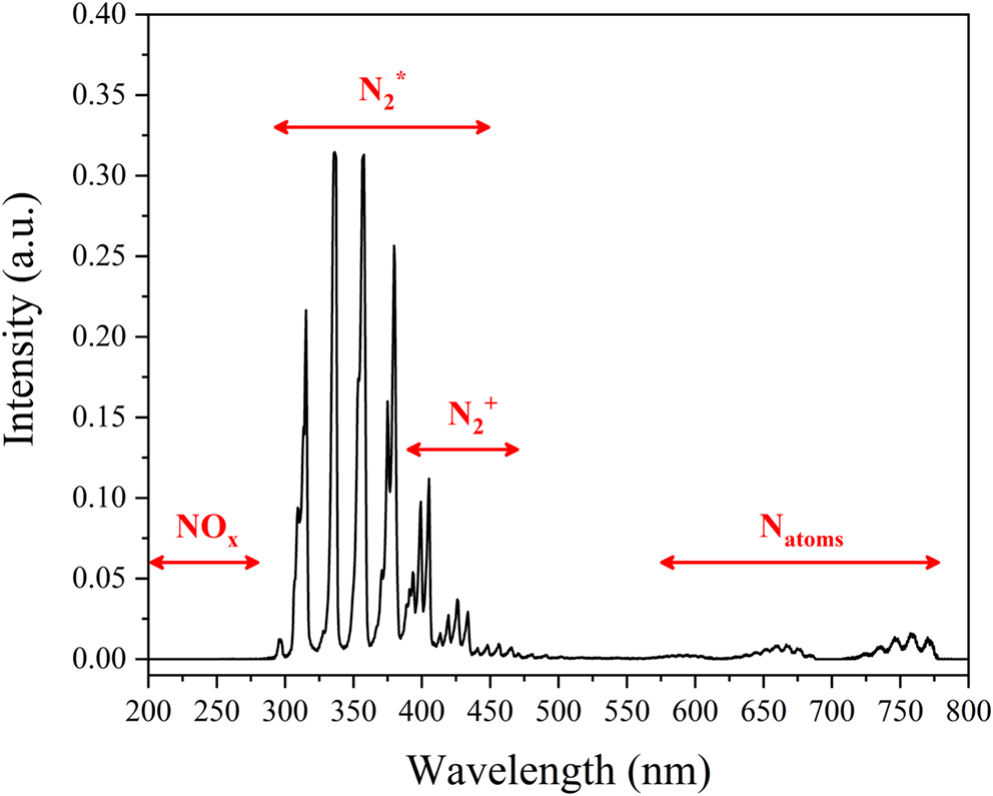
OES spectra of the N_2_ plasma in the P–L device. The operational parameters were set to a 2 mm gap distance, a capillary tube ID of 0.178 mm, a N_2_ flow rate of 30 mL min^–1^, and a discharge current of 2.0 mA.

The primary species detected include N_2_* (the second positive system of nitrogen (C^3^∏_u_ → B^3^∏_g_) in the UV), N_2_^+^ (first negative system of nitrogen (B^2^∑_u_^+^ → X^2^Π_g_^+^)) and N atoms.

### 3.8 Reaction Mechanism

Characterizing the chemistry and plasma-nanodroplets interface in full is highly challenging, and most conventional methods for analyzing these aspects, including the characteristics of aerosol, the transient species formed in the discharge and at the interface, and the nanodroplets and electrolyte, etc., cannot be applied to this specific case. On the other hand, we believe our experimental results could guide a mechanistic study to address this challenging topic, thereby enabling selectivity at least twice that of current electrocatalytic approaches for N_2_ to NH_3_ synthesis. Thus, in the following section, we discuss some mechanistic aspects to guide the rationalization of the data. However, these comments serve as a guide to better understand the complex chemistry present and to eventually further optimize, without claiming that the proposed mechanism is fully proven.

NTP offers a versatile approach to N_2_ activation via three primary mechanisms: electron-collision-induced excitation, dissociation, and ionization.^44–48^ Equations 8–10 represent the equations depicting the excitation and dissociation of N_2_, where each species exhibits distinct lifetimes and reactivities.^49^ The primary N_2_ species generated within the plasma include atomic nitrogen (N^•^) (eq 8), excited nitrogen species (N_2_*) (eq 9), and nitrogen ions (N_2_^+^) (eq 10).

$$e^{*}+N_{2}\to e+2N^{•}$$ 8

9 $$e^{*}+N_{2}\to e+N_{2}^{*}$$

10 $$e^{*}+N_{2}\to 2e+N_{2}^{+}$$

It is well-established that vibrationally excited nitrogen (N_2(ν)_) effectively reduces the activation barrier for the initial dissociative adsorption of N_2_, a crucial step in the catalytic production of NH_3_ from N_2_ and H_2_.^50^ For instance, Miyake et al.^47^ demonstrated that N_2(ν)_ plays a significant role in NH_3_ synthesis using atmospheric-pressure plasma, where the flux of N_2(ν)_ was over 4 orders of magnitude higher than that of atomic nitrogen (N^•^).

Hawtof et al.^17^ proposed a model for their P–L system in which N_2(ν)_ molecules are first dissolved into the liquid. Within the solution, these molecules react with H^•^, which are generated by H^+^ reduction facilitated by e_(aq)_^–^, leading to the formation of NH_3_ (NH_4_^+^), with N_2_H^•^ acting as an intermediary species. In a similar approach, Haruyama et al.^51^ suggested that NH^•^ is primarily formed at the plasma-water interface through reactions between N atoms and H_2_O molecules, after which it is further reduced in the liquid phase to NH_2_^•^ and NH_3_. Additionally, Pattyn et al.^19^ detected NH^•^ radicals in the discharge using OES measurements with a DC P–L device. On the other hand, Wang et al.^35^ described the presence of NH^•^ radicals when operating a gas–liquid interface pulsed discharge plasma device.

Building upon the reaction pathway proposed by Christianson et al.,^52^ and supported by subsequent research,^18,35,45^ a synthesis pathway for the reduction of N_2_ to NH_3_ within our DC P–L system can be proposed. Both H^•^ and N^•^ can be generated by plasma, according to eqs 5, 7 and 8, respectively. Sequentially, the first intermediate for NH_3_ synthesis (NH^•^ radical) is generated by several processes (eqs 11–14), both in the plasma (gas phase) and at the P–L interface.^19,51,53,54^

11 $$N_{\left(g\right)}^{•}+H_{\left(g\right)}^{•}\to {\mathrm{NH}}_{\left(g\right)}^{•}$$

12 $$N^{•}+H_{2}O\to {\mathrm{NH}}^{•}+{\mathrm{OH}}^{•}$$

13 $$N^{•}+{\mathrm{HO}}_{2}\to {\mathrm{NH}}^{•}+O_{2}$$

14 $$N^{•}+{\mathrm{OH}}^{•}\to {\mathrm{NH}}^{•}+O^{•}$$

N_2_H^•^ radical could also be generated by the initial addition of H^•^ at the P–L interface to N_2(ν)_, according to eq 15:^17,52^

15 $$N_{2\left(\nu \right)}+H^{•}\to N_{2}H^{•}$$

NH^•^ radical, can then undergo further H^•^ and OH^•^ addition to generate NH_3_ (eqs 16–17), which is in our case trapped as NH_4_^+^ directly in the bulk solution (eq 18):

16 $${\mathrm{NH}}^{•}+2H^{•}\to {\mathrm{NH}}_{3\left(\mathrm{aq}\right)}$$

17 $${\mathrm{NH}}^{•}+2{\mathrm{OH}}^{•}\to {\mathrm{NH}}_{3\left(\mathrm{aq}\right)}+2O^{•}$$

18 $${\mathrm{NH}}_{3\left(\mathrm{aq}\right)}+H_{\left(\mathrm{aq}\right)}^{+}\to {{{\mathrm{NH}}_{4}}^{+}}_{\left(\mathrm{aq}\right)}$$

The lack of an observable signal for the NH^•^ species (A^3^∏ → X^3^∑^–^) in the 336–337 nm region^19,35,55,56^ strongly supports the assumption that this critical intermediate is generated directly at the P–L interfaces. This observation aligns with the mechanism proposed by Hawtof et al.^17^ for H^•^ addition to N_2(ν)_ at the P–L interface (eq 15), rather than in the plasma gas phase.

OH^•^ signal (A^2^∑^+^ → X^2^∏) is present in the 309 nm region as illustrated in Figure S6.^24,35,36^ This radical can be generated by electron-impact dissociation of H_2_O as depicted in eq 7, but also from reaction in eqs 19–21:^16,54^

19 $$2N^{•}+H_{2}O\to N_{2}+2{\mathrm{OH}}^{•}+H^{•}$$

20 $$O^{*}+H_{2}O\to {\mathrm{OH}}^{•}+{\mathrm{OH}}^{•}$$

21 $${N_{2}}^{*}+H_{2}O\to N_{2}+H^{•}+{\mathrm{OH}}^{•}$$

Although OH^•^ participates in the generation of important intermediate species such as NH^•^, NH_2_^•^, and NH_3_ (eqs 14 and 17), they also have detrimental effects on NH_3_/NH_4_^+^ formation. Under NTP conditions, OH^•^ can trigger reverse reactions leading to the decomposition of NH^•^, NH_2_^•^, and NH_3_ (eqs 22–25).^35,54^

22 $${\mathrm{OH}}^{•}+{\mathrm{NH}}^{•}\to H_{2}+\mathrm{NO}$$

23 $${\mathrm{OH}}^{•}+{\mathrm{NH}}^{•}\to H^{•}+\mathrm{HNO}$$

24 $${\mathrm{OH}}^{•}+{{\mathrm{NH}}_{2}}^{•}\to H_{2}O+{\mathrm{NH}}^{•}$$

25 $${\mathrm{OH}}^{•}+{\mathrm{NH}}_{3}\to H_{2}O+{{\mathrm{NH}}_{2}}^{•}$$

It is worth noting that, although no NO_*x*_ species were generated in the gas phase during our tests (Figures 4 and S6), DC-driven P–L systems can produce nitrate (NO_3_^–^) and nitrite (NO_2_^–^) ions in solution, as documented in the literature^17,19^ and confirmed in this work by ion chromatography analysis. Both species act as chemical scavengers for e_(aq)_^–^,^17,23,26,29^ with NO_3_^–^ exhibiting a rate coefficient of 7.0 ± 2.6 × 10^9^ M^–1^ s^–1^ and NO_2_^–^ exhibiting a similar coefficient of 5.2 ± 2.6 × 10^9^ M^–1^ s^–1^. However, NO_2_^–^ reacts approximately 500 times faster with H^•^ than NO_3_^–^.^17^ The reactions of NO_3_^–^ and NO_2_^–^ with e_(aq)_^–^ generate various radical species, (NO_3_^•^)^2–^ and (NO_3_H^•^)^2–^ in the case of NO_3_^–^, (NO_2_^•^)^2–^ and NO^•^ in the case of NO_2_^–^.^17,23,26^ Therefore, the presence of NO_3_^–^ and NO_2_^–^ in the electrolyte solution during plasma operation cannot be considered as nitrogen sources for NH_3_ synthesis.

To evaluate whether dissolved NO_*x*_^–^ species contribute to NH_3_ formation, rigorous control experiments were performed using aqueous NO_3_^–^ and NO_2_^–^ solutions prepared in 0.25 mM H_2_SO_4_ at fixed concentrations. Based on concentrations measured under standard operating conditions, solutions containing 100 ppm of NO_3_^–^ and 30 ppm of NO_2_^–^ were prepared (see SI *Section 1.3* for details). Each solution was independently subjected to Ar plasma treatment for 30 min under conditions identical to those used in Figure 3c. In all cases, no measurable increase in NH_4_^+^ concentration was detected following plasma exposure. These results demonstrate that plasma-generated NO_*x*_^–^ species in the liquid phase do not act as nitrogen precursors for NH_3_ synthesis in the D-C driven P–L hybrid system.

Note additionally that tests using N_2_ + O_2_ mixtures rather than N_2_ only to promote the generation of NO_*x*_, according to the known NTP mechanism, fail in enhancing NH_3_ formation, thus indirectly proving that in our case, the high FE to NH_3_ is due to the presence of a direct mechanism of hydrogenation of N_2_ rather than a mechanism passing through NO_*x*_ formation. On the other hand, passing through them would require a higher consumption of protons/electrons, and it is thus not a rational objective.

Our experimental results on the roles of operational parameters and device configuration highlight the critical role of aerosol formation at the P–L interface, identifying it as the preferred reaction region. Equations 15–17 could thus describe the processes occurring at the surface of aerosol nanodroplets, which serve as the primary site for the direct synthesis of NH_3_ from N_2_.

### 3.9 Role of Water Nanodroplets in the Plasma-Induced Aerosol

As discussed in the specific results on the effect of device configuration and operational parameters on performance in the direct N_2_-to-NH_3_ conversion, the experimentally observed behavior cannot be rationalized without considering a primary reaction region, namely, the surface of the nanodroplets generated by the interaction of the *micro*-discharge with the liquid electrolyte. While the role of aerosol in this reaction has been previously proposed,^22^ it has not been proven that its formation is strongly dependent on the device configuration and operational parameters. Thus, the ability to control and tune these parameters becomes crucial for improving performance, particularly in key areas such as PR, FE, and YI. We attempt to improve performance by flowing an aerosol (externally generated) in proximity to the *micro*-discharge area. However, this introduces significant stability problems in the *micro*-discharge (Figure S4), resulting in poor performances, with an NH_3_ FE of 11.3% and a PR of 0.05 mg h^–1^, which prevents verification of the improvements. The question remains open because it should first identify how to intensify local aerosol in the *micro*-discharge area without affecting the *micro*-discharge itself.

To further strengthen the experimental basis for this interpretation, we introduced a simplified surrogate system based on a dielectric barrier discharge (DBD) reactor, in which a continuous aerosol flux was injected (Figure S7). The device consists of a jet nebulizer, operating via the Venturi effect, to generate H_2_O droplets using N_2_ as the feed gas. The resulting H_2_O aerosol/N_2_ mixture was then introduced into a quartz tube, where it was activated by a plasma generated through the application of an alternating current (AC) voltage of 5 kV at a frequency of 47.5 kHz between a stainless-steel coaxial inner electrode and a grounded electrode composed of copper gauze wrapped around the quartz tube. Despite the different nature of the plasma reactor, the presence of aerosol led to a clear enhancement in the NH_3_ production rate (∼0.8 mg h^–1^) compared to aerosol-free operation, directly evidencing the beneficial role of microdroplets in plasma-assisted nitrogen fixation.

Although further studies are needed to consolidate these findings and, especially, to unravel the complex reaction mechanisms at the P–L interface, the present results offer valuable insights into how to enhance the performance of the plasma-water electrode *micro*-discharge interface and identify key factors for future research. Our results demonstrate that systematic engineering and optimization of the key operational parameters of the P–L device can significantly enhance both the FE and the PR. In particular, this optimization results in approximately a 3-fold increase in FE and a 2-fold improvement in PR. Our results also show the relevance of aerosol in the *micro*-discharge region, even though its direct measurement is not possible using aerosol measurement methods, as discussed earlier. On the other hand, increasing the nanodroplets concentration by feeding a water aerosol directly results in destabilizing the discharge, as previously commented. Therefore, to scale up the device, a different reactor configuration should be developed that maximizes aerosol in the discharge region while maintaining a stable discharge.

### 3.10 Application Prospects

Achieving high N_2_-to-NH_3_ performance in P–L systems has historically been a challenge.^57,58,13^ Due to differences in operating conditions and device types, the comparison is difficult. Additionally, performances are not stable and tend to change over time. Within the limitations of the literature data, as previously noted, it is nevertheless worthwhile to conduct this analysis and compare it with our results. A summary of literature N_2_-to-NH_3_ PR, N_2_ flow rate, and YI data with respect to our results is provided in Table 1. Note that while the N_2_ flow rate largely influences PR, the value of YI is preferable to analyze the specific efficiency of the mechanism of producing NH_3_.

**Table 1:** Comparative Summary of NH_3_/NH_4_^+^ Production and Conversion Rates Achieved by N_2_ Plasma Interacting with H_2_O

P–L system	**NH**_**3**_ **/NH**_**4**_^**+**^ **PR** (mg h^**–1**^ **)**	**N**_**2**_ **flow rate** (mL min^**–1**^ **)**	**N**_**2**_ **-to-NH**_**3**_ **/NH**_**4**_^**+**^ **YI (%)**	**refs**
DC	0.255	30	9.6 × 10^–3^	this work
DC	0.440	60^a^	8.3 × 10^–3^	^17^
DC	1.061	200	6.0 × 10^–3^	^18^
DC		1000	2.4 × 10^–5^ ^b^	^19^
AC plasma jet	0.124	200	7.0 × 10^–4^	^22^
AC plasma jet	0.936	1000	1.1 × 10^–3^	^24^
AC plasma discharges	1.182	1400	1.0 × 10^–3^	^59^
AC plasma jet + UV source	1.520	1000	1.7 × 10^–3^	^23^
AC plasma jet + UV source	0.143	3000	3.0 × 10^–4^	^46^
AC pulsed discharges	3.271	500	7.8 × 10^–3^	^55^
AC plasma jet	0.184	5000	4.4 × 10^–5^	^31^
AC spray-type plasma jet + UV source	2.710	57,000	5.4 × 10^–5^	^60^

a From US patent N° US20230286820A1.^61^

b Considering also NO_*x*_ as products.

Our optimized device achieved a superior YI value, combined with good PR and FE (70.5%). This result positions our device among the best reported for both DC and AC P–L systems. Notably, this achievement was achieved with a lower N_2_ flow rate than in other studies. This highlights the effectiveness of our engineering parameter optimization, which not only improved NH_3_ PR and FE but also minimized the required plasma gas feed. This reduction has a positive impact on the process’s overall environmental footprint.

Another essential aspect to consider is the device’s energy efficiency. The *breakdown voltage* required to generate plasma depends on several factors, including the gas type, pressure, and applied current. When the current is fixed and relatively low, the breakdown voltage tends to be higher for several reasons. Plasma generation requires sufficient energy to ionize the gas, meaning that electrons need to be accelerated to high enough energies to ionize gas atoms or molecules (the ionization process). With a lower current, fewer electrons are available to participate in the ionization process; thus, a higher voltage is needed to create a strong enough electric field to accelerate the electrons to ionizing energies. Furthermore, at low current, the number of collisions between electrons and gas molecules decreases. This reduces the ionization rate, requiring a higher voltage to overcome the ionization threshold and sustain the plasma.^33^

Figure 5 shows the experimental relationships among the aspects discussed above, indicating that the plasma’s operational voltage increases with decreasing fixed discharge current. Notably, energy consumption remained reasonably constant from 1.0 to 2.0 mA, increased at 2.5 mA, and remained nearly stable thereafter.

**Figure 5:**
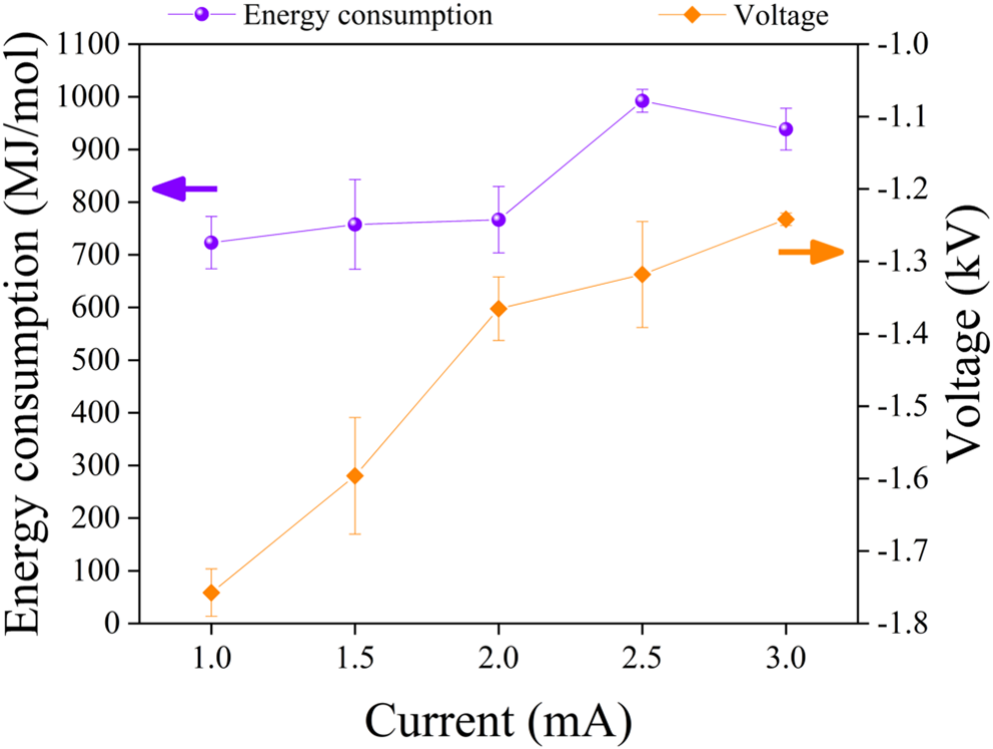
NH_3_ energy consumption and plasma operational voltage in the plasma-electrolytic system as a function of plasma discharge current. The complete data set is reported in the SI (Table S5b).

## 4 Conclusions

The optimized hybrid plasma–liquid (P–L) electrolytic system for sustainable NH_3_ synthesis from N_2_ and H_2_O at ambient temperature and pressure achieves a maximum production rate (PR) of 0.255 mg h^–1^ and a maximum Faradaic efficiency (FE) of 70.5%. The optimized system also demonstrates a high N_2_-to-NH_3_ instantaneous yield (YI) of 9.6 × 10^–3^%, outperforming previously reported P–L configurations.

This study demonstrated that stable operation and high performance are critically dependent on the systematic optimization of key engineering parameters. These parameters include plasma gap distance, gas feed flow rate, plasma electrode inner diameter, and electrolyte management (specifically, saturation time and stirring).

The analysis of these key engineering aspects, supported by insights from OES analysis, provides relevant guidance not only for enhancing performance but also for understanding the fundamental role of plasma-induced aerosol generation in the design of such devices. The surface of nanodroplets formed at the P–L interface is a critical region to promote the direct synthesis of NH_3_, where vibrationally excited N_2_ molecules (N_2(ν)_) react with hydrogen radicals (H^•^), generated by the interaction of hot plasma electrons with water. On the other hand, the side reaction of H^•^ recombination to form H_2_ molecules lowers the FE, highlighting the delicate balance between maximizing aerosol formation and reactive species generation, while tuning fluid-dynamic conditions to favor the formation of NH^•^, a precursor to NH_3_. Thus, there is a complex dependence on operating parameters and device design. The data reported in this work shed light on effective strategies for further improvement and offer guidance for system engineering to scale up the technology to industrial-relevant reactor and operational conditions.

While further investigation is necessary to elucidate the underlying mechanisms fully, these results highlight the potential of this sustainable plasma-assisted approach for N_2_ fixation under ambient conditions, particularly for decentralised, small-scale NH_3_ production. An engineering redesign is nevertheless required for scaling up technology.

Proving the outlined mechanism and role of nanodroplets at the interface is extremely challenging. Current methods to characterize aerosol in the discharge region and at the interface with the electrolyte, as well as to analyze in detail the transient species formed in the discharge region, in nanodroplets, and at the interface between the discharge and the electrolyte, do not provide reliable indications to support the proposed mechanism. We are developing a chemical reaction model based on quantum-mechanical simulations to rationalize plasma–liquid interfacial chemistry and gain deeper insights into the effective role of *micro*-droplets, but this will be the objective of a future dedicated paper, as it is extremely challenging.

This study focuses on demonstrating how system performance can be optimized through systematic engineering and careful operational management of a plasma–liquid hybrid device. Nevertheless, it provides very useful experimental support for mechanistic and theoretical modeling. Thus, it will be a strong push toward mechanistic studies, which, however, are more challenging than usual. Thus, they need guidance based on experimental evidence, which is what our paper aims to provide.

## Supplementary Material



## Notes

Additional experimental details, materials, and methods. Schematic representation of the hybrid plasma-electrolytic system. Calibration curves for NH_4_^+^, NO_3_^–^, and NO_2_^–^. Standard voltage and current waveforms. Equations used to calculate Faradaic efficiency (FE), production rate (PR), instantaneous N_2_-to-NH_3_ yield (YI), and energy consumption (EC). Current waveform during external aerosol generation. Hydrogen production as a function of time. Additional optical emission spectroscopy (OES) spectra. Dielectric-barrier discharge (DBD) reactor. Tables of data. Electrolyte saturation considerations (PDF)

## List of Acronyms

P–L: plasma–liquid
NTP: nonthermal plasma
FE: Faradaic efficiency
PR: production rate
YI: instantaneous yield
ID: inner diameter
HER: hydrogen evolution reaction
OER: oxygen evolution reaction
OES: optical emission spectroscopy
BT: blank test
