# Supplementary material for: Engineering Plasma–Liquid Microdischarge Systems for Direct N2‑to-NH3 Conversion at Ambient Conditions
Source: ACS Sustain Chem Eng. 2026 Mar 14;14(12):6062–74. doi: 10.1021/acssuschemeng.5c13108 (PMC13040528; doi:10.1021/acssuschemeng.5c13108)
Supplement: Supplementary file 1 [file sc5c13108_si_001.pdf]

Supporting information

Engineering plasma-liquid *micro*-discharge systems for direct N_2-to-NH_3 conversion at ambient conditions

Marco Francesco Torre[1], Lavanya Veerapuram[1], Francesco Tavella[1,], Chiara Genovese[1], Siglinda Perathoner[1], Federica Torrigino[2], Pierdomenico Biasi[2], Gabriele Centi[1], Claudio Ampelli[1]*

1 Department of Chemical, Biological, Pharmaceutical and Environmental Sciences (ChiBioFarAm), University of Messina, ERIC aisbl and CASPE/INSTM, Viale Ferdinando Stagno d'Alcontres 31, 98166 Messina, Italy.

2 Basic Research Department, Casale SA, Via G. Pocobelli 6, 6900 Lugano, Switzerland.

* corresponding author

E-mail: francesco.tavella@unime.it

Number of pages: 15

Number of figures: 7

Number of tables: 7

Section 1

Materials, experimental procedures, and NH_3 measurement

1.1. Materials

The plasma-electrolytic device (Figure S1) consists of a quartz glass reaction cell (50 mL) sealed with a Teflon lid featuring four openings. Two of these openings are used for gas tubes (inlet and outlet), one for the insertion of a stainless-steel capillary tube (outer diameter: 1.59 mm, inner diameter: 0.178 – 0.254 – 0.508 mm, length: 10 cm; Restek) for plasma generation, and the last one for the insertion of a Pt plate electrode. The capillary tube is connected to a mass-flow controller (MFC) to regulate the gas flow rate. N_2 (99.9999%, Sol Group) continuously flows inside the capillary tube, positioned at a specific distance above the electrolytic solution, to generate plasma when a potential is applied between the two electrodes, using a high-voltage power supply (MPL200 Series, XP Power). The electrolyte solution consisted of a 0.25 mM H_2SO_4 solution (96%, Carlo Erba) in Milli-Q water (18.2 MΩ). The system operates in direct current (DC) and galvanostatic mode. It includes two resistors: a 1.0 MΩ ballast resistor (R_b) in series to control current peaks during plasma ignition, and a 500 Ω resistor (R_i) in series between the Pt anode and ground, used to measure the current from the voltage drop across it. The plasma operational voltage was measured using a high-voltage probe (CT4432, Cal Test Electronics) between the R_b and the plasma cathode, which was connected to an oscilloscope (2204A, Pico Technology). Optical emission spectroscopy (OES) spectra of the plasma were recorded using an ILT 950 spectrometer (International Light Technologies) over 200-800 nm, with an optical fiber placed close to the quartz cell. Data are presented as mean ± σ (standard deviation), calculated from the average of three independent tests conducted under identical operational conditions.

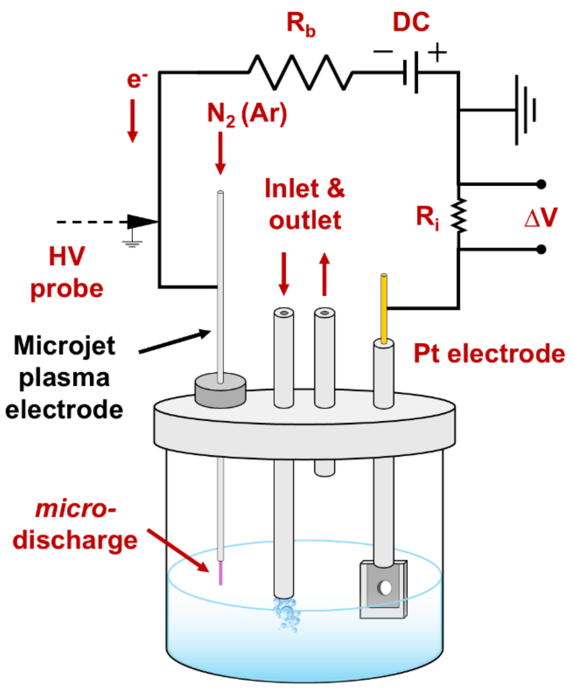

Figure S1. Detailed representation of the hybrid plasma-electrolytic system.

1.2. Measurement of NH_3 (NH_4^+), NO_3^- and NO_2^- produced

The production of NH_3, NO_3^-, and NO_2^- in the plasma-electrolytic system was evaluated using ion chromatography, which measured the concentrations of ammonium ions (NH_4^+), nitrate ions (NO_3^-), and nitrite anions (NO_2^-) in the solution. The ion chromatograph (Eco IC, Metrohm) was equipped with a cation column (Metrosep C6 – 150/4.0, Metrohm) for NH_4^+ analysis and an anion column (Metrosep A Supp 5 – 250/4.0) for NO_3^- and NO_2^- analysis.

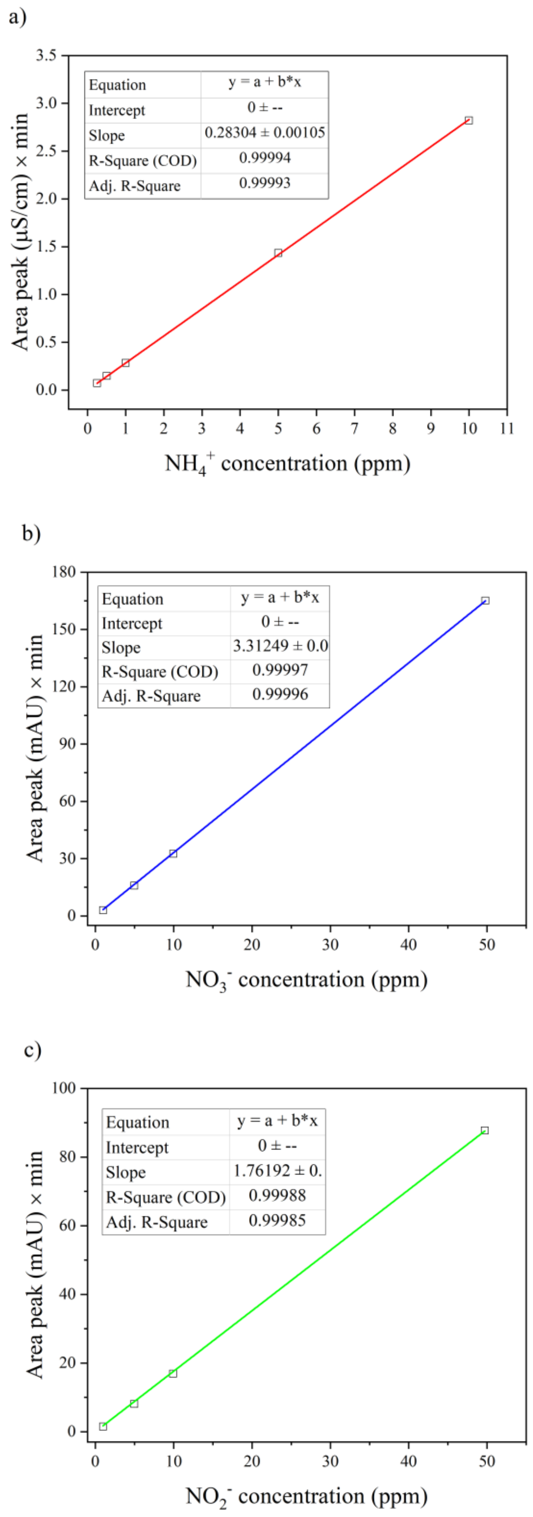

Figure S2. ion chromatography calibration curve for a) NH_4^+, b) NO_3^- and c) NO_2^- determination.

1.3. Experimental procedures

The standard procedure for all tests was as follows: to evaluate the presence of NH_3 in the ambient air, a blank test (BT) was performed before each test. A BT involves purging the electrolyte solution with N_2 at a flow rate of 50 mL min^{-1} for 15 minutes to mimic the saturation time of each primary test. After 15 minutes, the solution was analysed by IC to determine the eventual concentration of NH_4^+. Before igniting the plasma, a 20 mL solution was bubbled with N_2 for 15 minutes at a flow rate of 50 mL min^{-1} to eliminate any dissolved gases and purge the headspace of the glass cell. After 15 minutes, the voltage was increased until plasma ignition occurred, and each test was run for 30 minutes.

For experiments conducted with fixed concentrations of NO_3^- and NO_2^-, the experimental procedure followed that used for the standard tests. For the BT step, the electrolyte solution was purged with Ar at a flow rate of 50 mL min^{-1} for 30 minutes. After purging, the solution was analyzed by IC to determine the background NH_4^+ concentration. Subsequently, a 20 mL aliquot of the solution was again bubbled with Ar at a flow rate of 50 mL min^{-1} for 30 minutes, after which the Ar flow rate was reduced to 30 mL min^{-1} to initiate plasma ignition. Each experiment was conducted for 30 minutes under otherwise identical operating conditions.

1.4. Data acquisition and Calculations

The exact total operation time for each test was determined by analysing the oscilloscope trace, which included both the electrical voltage between the electrodes and the current measured across the resistor R_i. Faradaic efficiency (FE) was determined assuming a three-electron reaction ($\frac{1}{2}N_2 + 3H^+ + 3e^- \rightarrow NH_3$). Although the applied current was set to be constant, minor fluctuations occurred throughout the experiments due to plasma instabilities, particularly during plasma ignition (Figure S3). These fluctuations, while rapid on the order of μs, did not affect the average current over the experimental duration. To ensure accurate faradaic NH_4^+ calculations, the current during each test was recorded and integrated over time to obtain the total charge, $Q = \int I(t)dt$.

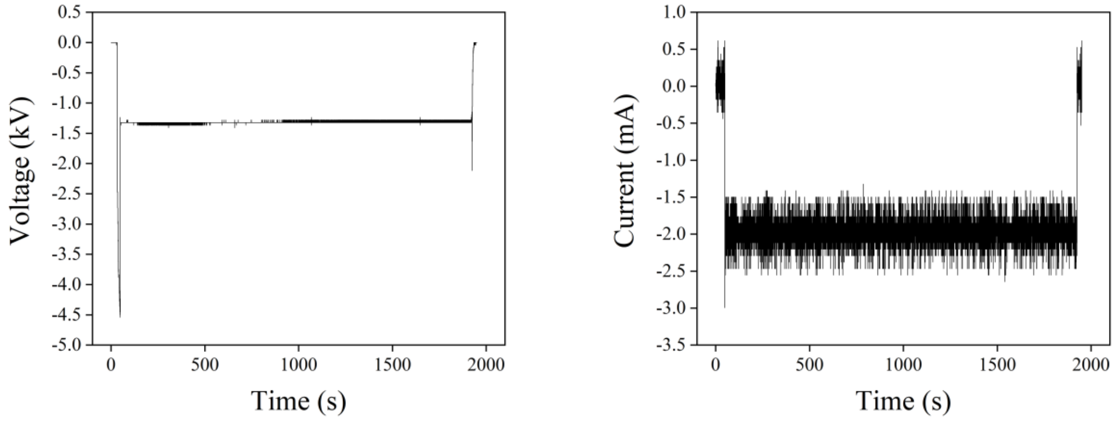

Figure S3. voltage (left) and current (right) waveforms measured during NH_3 synthesis in the plasma-electrolytic system. Sampling time was set to 20 μs. Waveforms refer to run N°3 of **Table S3** "capillary tube ID 0.178 mm".

Faradaic efficiency (FE) was calculated as follows:

$$FE(\%) = \frac{n \times z \times F}{Q} \times 100\%$$

Where n represents the number of moles of the product (NH_4^+) measured by IC, z is the number of electrons (3 for the NH_3 reaction), F is Faraday's constant (9.64853×10^4 C mol^{-1}), and Q (A × s) is the total charge.

Overall productivity (PR) was calculated as follows:

$$Overall\ Productivity\ \left(\frac{mg}{h}\right) = \frac{C_{NH_4^+} \times \mathrm{V} \times 60\ min.}{t}$$

where $C_{NH_4^+}$ (mg L^{-1}) is the experimentally measured concentration of NH_4^+ by IC, V (L) is the volume of the solution, and t (min.) is the exact time of the test acquired by the oscilloscope.

The N_2-to-NH_3 (NH_4^+) instantaneous yield (YI) was calculated as the ratio of NH_4^+ produced to the amount of injected N_2 (averaged in 30 minutes) at the selected flow rate:

$$N_2 - to - NH_4^+\ instantaneous\ yield\ (\%) = \frac{NH_4^+ mol\ produced/2}{N_2\ mol\ injected} \times 100\%$$

Energy consumption was calculated as follows:

$$Energy\ consumption\ \left(\frac{MJ}{mol}\right) = \frac{V \times I}{(PR/10^6)} \times MM_{NH_4^+} \times 3.6 \times 10^{-3}$$

Where V (kV) and I (A) are the plasma voltage and current in the plasma-electrolytic system, PR is the NH_4^+ overall productivity (mg h^{-1}) and $MM_{NH_4^+}$ (g mol^{-1}) is the molar mass of NH_4^+.

1.5. Current waveform with an external water aerosol

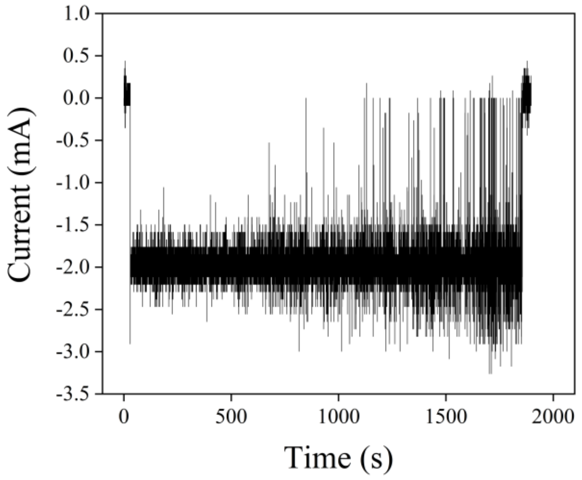

Figure S4. Current recorded during NH_3 synthesis in the P-L system with an external water aerosol. The operational conditions were set to a 2 mm gap distance, capillary tube ID of 0.178 mm, N_2 flow rate of 30 mL min^{-1}, and a discharge current of 2.0 mA.

1.6. Hydrogen (H_2) measurement

The quantification of hydrogen in the gas streams was performed using an online micro gas chromatograph (MicroGC GCX, Pollution Analytic Equipment). The analysis utilised a molecular sieve column with N_2/Ar as the carrier gas.

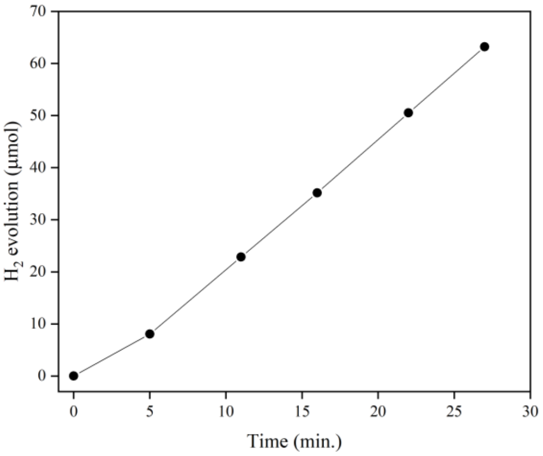

Figure S5. H_2 evolution as a function of time in the plasma-electrolytic device operated at a fixed plasma discharge current of 2.0 mA.

1.7. OES spectrum

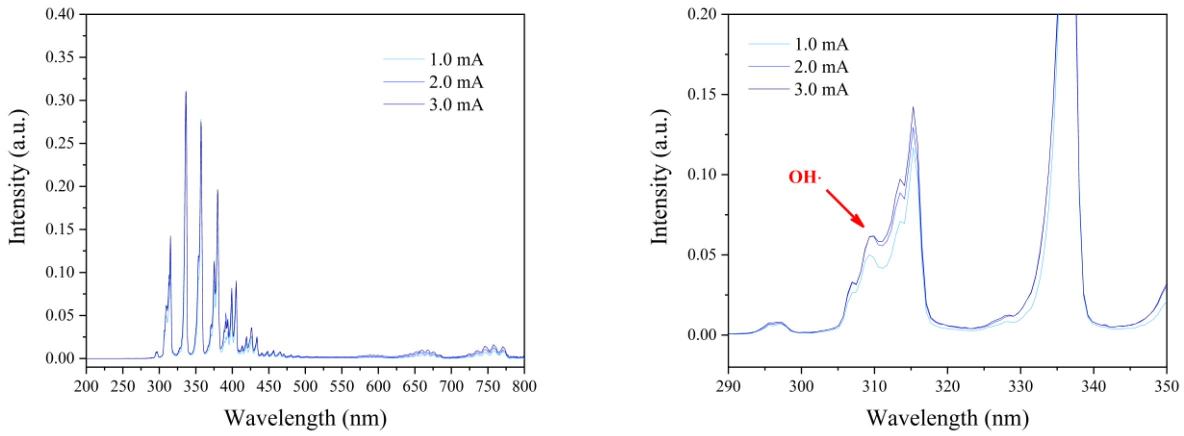

Figure S6. OES spectra of the N_2 plasma in the P-L device. The operational parameters were set to a 2 mm gap distance, a capillary tube ID of 0.178 mm, a N_2 flow rate of 30 mL min^{-1}, and a discharge current of 1.0, 2.0 and 3.0 mA.

1.8. Dielectric-barrier discharge (DBD) reactor for plasma-aerosol experiment

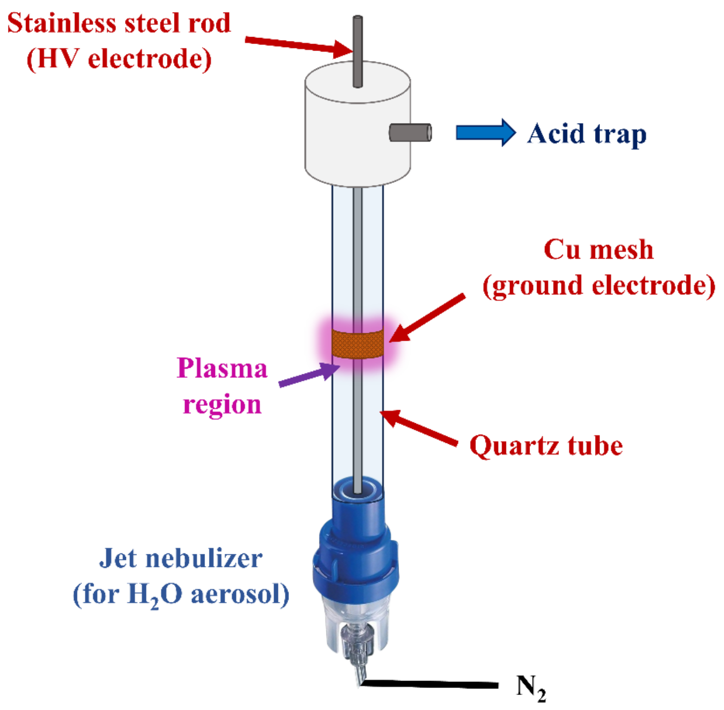

Figure S7. DBD reactor for plasma-aerosol experiment. The operational parameters were set to a 1.5 mm gap distance, and operational voltage of 5 kV at a frequency of 47.5 kHz between the stainless-steel HV coaxial inner electrode and the grounded electrode.

Section 2

Complete set of data for all experiments

Table S1. Effect of gap distance, between the capillary tube and the electrolyte solution surface, on NH_3 production rate and faradaic efficiency. Ion chromatography was employed to evaluate NH_3 production in the plasma-electrolytic system by quantifying the concentration of NH_4^+ formed in the solution. Data are reported as average ± σ.

Gap distance = 1 mm

Run	NH_4^+ produced (mg L^{-1})	NH_4^+ produced (mg)	Production rate (mg h^{-1})	Faradaic efficiency (%)
1	1.791	0.0358	0.072	16.3
2	1.589	0.0318	0.064	14.5
3	1.964	0.0393	0.076	17.4
**Average**			**0.071 ± 0.007**	**16.1 ± 1.5**

Gap distance = 2 mm

Run	NH_4^+ produced (mg L^{-1})	NH_4^+ produced (mg)	Production rate (mg h^{-1})	Faradaic efficiency (%)
1	1.316	0.0263	0.071	16.1
2	5.994	0.0120	0.120	27.2
3	2.729	0.0546	0.109	24.8
**Average**			**0.100 ± 0.026**	**22.7 ± 5.9**

Gap distance = 3 mm

Run	NH_4^+ produced (mg L^{-1})	NH_4^+ produced (mg)	Production rate (mg h^{-1})	Faradaic efficiency (%)
1	2.708	0.0542	0.117	25.8
2	1.044	0.0209	0.044	10.0
3	1.968	0.0394	0.079	17.9
**Average**			**0.080 ± 0.037**	**17.9 ± 7.9**

Table S2. Effect of N_2 flow rate on NH_3 production rate and faradaic efficiency. Ion chromatography was employed to evaluate NH_3 production in the plasma-electrolytic system by quantifying the concentration of NH_4^+ formed in the solution. Data are reported as average ± σ. Refers to **Table S1** "gap distance 2 mm" for data at 50 mL min^{-1}.

Flow rate = 20 mL min^{-1}

Run	NH_4^+ produced (mg L^{-1})	NH_4^+ produced (mg)	Production rate (mg h^{-1})	Faradaic efficiency (%)
1	0.834	0.0167	0.034	7.8
2	3.045	0.0609	0.121	27.4
3	2.901	0.0580	0.115	26.3
**Average**			**0.090 ± 0.049**	**20.5 ± 11.0**

Flow rate = 30 mL min^{-1}

Run	NH_4^+ produced (mg L^{-1})	NH_4^+ produced (mg)	Production rate (mg h^{-1})	Faradaic efficiency (%)
1	3.987	0.0797	0.157	35.7
2	4.313	0.0863	0.167	38.1
3	4.714	0.0943	0.187	42.5
**Average**			**0.170 ± 0.015**	**38.8 ± 3.5**

Flow rate = 40 mL min^{-1}

Run	NH_4^+ produced (mg L^{-1})	NH_4^+ produced (mg)	Production rate (mg h^{-1})	Faradaic efficiency (%)
1	1.879	0.0376	0.075	17.0
2	2.929	0.0586	0.116	26.4
3	1.414	0.0283	0.056	12.7
**Average**			**0.082 ± 0.031**	**18.7 ± 7.0**

Table S3. Effect of capillary tube inner diameter (ID) on NH_3 production rate and faradaic efficiency. Ion chromatography was employed to evaluate NH_3 production in the plasma-electrolytic system by quantifying the concentration of NH_4^+ formed in the solution. Data are reported as average ± σ. Refers to **Table S2** "flow rate 30 mL min^{-1}" for data at 0.508 mm ID.

Capillary tube ID = 0.178 mm

Run	NH_4^+ produced (mg L^{-1})	NH_4^+ produced (mg)	Production rate (mg h^{-1})	Faradaic efficiency (%)
1	5.876	0.0118	0.228	52.0
2	5.382	0.0108	0.211	48.1
3	6.259	0.0125	0.240	54.8
**Average**			**0.226 ± 0.015**	**51.6 ± 3.3**

Capillary tube ID = 0.254 mm

Run	NH_4^+ produced (mg L^{-1})	NH_4^+ produced (mg)	Production rate (mg h^{-1})	Faradaic efficiency (%)
1	1.680	0.0336	0.067	15.2
2	1.356	0.0271	0.075	12.2
3	1.777	0.0355	0.078	15.3
**Average**			**0.073 ± 0.006**	**14.2 ± 11.7**

Table S4. Effect of electrolyte solution saturation time on NH_3 production rate and faradaic efficiency. Ion chromatography was employed to evaluate NH_3 production in the plasma-electrolytic system by quantifying the concentration of NH_4^+ formed in the solution. Data are reported as average ± σ. Refers to **Table S3** "Capillary tube ID = 0.178 mm" for data with electrolyte solution saturation time of 15 minutes.

Saturation time = 30 min.

Run	NH_4^+ produced (mg L^{-1})	NH_4^+ produced (mg)	Production rate (mg h^{-1})	Faradaic efficiency (%)
1	5.019	0.0100	0.198	45.1
2	5.807	0.0116	0.227	51.9
3	6.016	0.0120	0.236	53.8
**Average**			**0.220 ± 0.020**	**50.3 ± 4.5**

Saturation time = 45 min.

Run	NH_4^+ produced (mg L^{-1})	NH_4^+ produced (mg)	Production rate (mg h^{-1})	Faradaic efficiency (%)
1	5.336	0.0107	0.209	47.7
2	5.155	0.0103	0.203	46.4
3	5.830	0.0117	0.232	52.8
**Average**			**0.215 ± 0.015**	**49.0 ± 3.4**

Saturation time = 60 min.

Run	NH_4^+ produced (mg L^{-1})	NH_4^+ produced (mg)	Production rate (mg h^{-1})	Faradaic efficiency (%)
1	6,042	0.0121	0,237	54,2
2	5,061	0.0101	0,199	45,3
3	6,043	0.0121	0,237	54,1
**Average**			**0.224 ± 0.022**	**51.2 ± 5.1**

Table S5a. Effect of discharge current on NH_3 production rate and faradaic efficiency. Ion chromatography was employed to evaluate NH_3 production in the plasma-electrolytic system by quantifying the concentration of NH_4^+ formed in the solution. Data are reported as average ± σ. Refers to **Table S3** "Capillary tube ID = 0.178 mm" for data at 2.0 mA.

Discharge current = 1.0 mA

Run	NH_4^+ produced (mg L^{-1})	NH_4^+ produced (mg)	Production rate (mg h^{-1})	Faradaic efficiency (%)
1	4.036	0.0807	0.157	74.8
2	3.716	0.0743	0.147	69.9
3	3.530	0.0706	0.141	66.8
**Average**			**0.148 ± 0.008**	**70.5 ± 4.0**

Discharge current = 1.5 mA

Run	NH_4^+ produced (mg L^{-1})	NH_4^+ produced (mg)	Production rate (mg h^{-1})	Faradaic efficiency (%)
1	5.288	0.0106	0.209	62.8
2	5.358	0.0107	0.213	64.2
3	4.841	0.0968	0.187	56.6
**Average**			**0.203 ± 0.014**	**61.2 ± 4.1**

Discharge current = 2.5 mA

Run	NH_4^+ produced (mg L^{-1})	NH_4^+ produced (mg)	Production rate (mg h^{-1})	Faradaic efficiency (%)
1	5.271	0.0105	0.206	36.9
2	5.553	0.0111	0.220	39.6
3	5.392	0.0108	0.214	38.6
**Average**			**0.213 ± 0.007**	**38.4 ± 1.3**

Discharge current = 3.0 mA

Run	NH_4^+ produced (mg L^{-1})	NH_4^+ produced (mg)	Production rate (mg h^{-1})	Faradaic efficiency (%)
1	6.662	0.0133	0.264	39.8
2	6.084	0.0122	0.242	36.3
3	6.555	0.0131	0.259	38.9
**Average**			**0.255 ± 0.012**	**38.3 ± 1.8**

Table S5b. Effect of discharge current on NH_3 energy consumption, N_2-to-NH_4^+ conversion rate and plasma operational voltage. Data are reported as average ± σ.

Discharge current = 1.0 mA

Run	Voltage (kV)	Energy consumption (MJ mol^{-1})	N_2-to-NH_4^+ conversion rate (%)
1	-1.75	678.3	5.91×10^{-3}
2	-1.73	714.6	5.53×10^{-3}
3	-1.79	776.5	5.33×10^{-3}
**Average**	**-1.76 ± 0.03**	**723.1 ± 49.6**	**$5.59 \times 10^{-3} \pm 2.96 \times 10^{-4}$**

Discharge current = 1.5 mA

Run	Voltage (kV)	Energy consumption (MJ mol^{-1})	N_2-to-NH_4^+ conversion rate (%)
1	-1.51	697.2	7.87×10^{-3}
2	-1.60	721.0	8.02×10^{-3}
3	-1.68	855.0	7.05×10^{-3}
**Average**	**-1.60 ± 0.08**	**757.8 ± 85.1**	**$7.65 \times 10^{-3} \pm 5.24 \times 10^{-4}$**

Discharge current = 2.0 mA

Run	Voltage (kV)	Energy consumption (MJ mol^{-1})	N_2-to-NH_4^+ conversion rate (%)
1	-1.41	784.4	8.57×10^{-3}
2	-1.36	819.0	7.95×10^{-3}
3	-1.32	696.9	9.05×10^{-3}
**Average**	**-1.37 ± 0.04**	**766.7 ± 62.9**	**$8.52 \times 10^{-3} \pm 5.52 \times 10^{-4}$**

Discharge current = 2.5 mA

Run	Voltage (kV)	Energy consumption (MJ mol^{-1})	N_2-to-NH_4^+ conversion rate (%)
1	-1.23	967.8	7.74×10^{-3}
2	-1.37	1000.8	8.29×10^{-3}
3	-1.35	1008.5	8.07×10^{-3}
**Average**	**-1.32 ± 0.07**	**992.4 ± 21.6**	**$8.03 \times 10^{-3} \pm 2.75 \times 10^{-4}$**

Discharge current = 3.0 mA

Run	Voltage (kV)	Energy consumption (MJ mol^{-1})	N_2-to-NH_4^+ conversion rate (%)
1	-1.25	908.6	9.96×10^{-3}
2	-1.23	983.7	9.11×10^{-3}
3	-1.24	923.5	9.74×10^{-3}
**Average**	**-1.24 ± 0.01**	**938.6 ± 39.7**	**$9.60 \times 10^{-3} \pm 4.45 \times 10^{-4}$**

Table S6. Effect of electrolyte solution stirring on NH_3 production rate and faradaic efficiency. Ion chromatography was employed to evaluate NH_3 production in the plasma-electrolytic system by quantifying the concentration of NH_4^+ formed in the solution. Data are reported as average ± σ. Refers to **Table S3** "Capillary tube ID = 0.178 mm" for data without stirring.

Stirring ON = 100 rpm

Run	NH_4^+ produced (mg L^{-1})	NH_4^+ produced (mg)	Production rate (mg h^{-1})	Faradaic efficiency (%)
1	5.041	0.0101	0.200	45.4
2	5.648	0.0113	0.224	50.9
3	5.425	0.0109	0.214	48.8
**Average**			**0.213 ± 0.012**	**48.4 ± 2.8**

Section 3

Electrolyte saturation time calculations and considerations

As stated in the main text, the solubility of N_2 (in H_2O at 298.15 K under 1 atm air atmosphere) is 5.1×10^{-4} mol L^{-1}. Thus, to thoroughly saturate the volume of electrolyte solution fixed at 20 mL, a minimum amount of N_2 moles is required:

mol $N_2 = 5.1 \times 10^{-4}$ mol $L^{-1} \times 0.02$ L $= 1.02 \times 10^{-5}$ mol

By considering an N_2 purging flow rate of 10 mL min^{-1}, we can calculate the N_2 moles injected by applying the *Ideal gas law*:

$$pV = nRT \rightarrow n = \frac{p \times V}{R \times T}$$

$$n = \frac{1\ atm \times 0.01\ L/min}{0.0821 \frac{atm \times L}{K \times mol} \times 298.15\ K} = 4.08 \times 10^{-4}\ mol\ min^{-1}$$
